# Transcriptomic evidence for microbial sulfur cycling in the eastern tropical North Pacific oxygen minimum zone

**DOI:** 10.3389/fmicb.2015.00334

**Published:** 2015-05-11

**Authors:** Molly T. Carolan, Jason M. Smith, J. M. Beman

**Affiliations:** ^1^Life and Environmental Sciences and Sierra Nevada Research Institute, University of California at MercedMerced, CA, USA; ^2^Monterey Bay Aquarium Research InstituteMoss Landing, CA, USA

**Keywords:** sulfate reduction, sulfur oxidation, sulfur cyling, transcriptomics, oxygen minimum zone

## Abstract

Microbial communities play central roles in ocean biogeochemical cycles, and are particularly important in in oceanic oxygen minimum zones (OMZs). However, the key carbon, nitrogen, and sulfur (S) cycling processes catalyzed by OMZ microbial communities are poorly constrained spatially, temporally, and with regard to the different microbial groups involved. Here we sample across dissolved oxygen (DO) gradients in the oceans’ largest OMZ by volume—the eastern tropical North Pacific ocean, or ETNP—and quantify 16S rRNA and functional gene transcripts to detect and constrain the activity of different S-cycling groups. Based on gene expression profiles, putative dissimilatory sulfite reductase (*dsrA*) genes are actively expressed within the ETNP OMZ. *dsrA* expression was limited almost entirely to samples with elevated nitrite concentrations, consistent with previous observations in the Eastern Tropical South Pacific OMZ. *dsrA* and ‘reverse’ dissimilatory sulfite reductase (*rdsrA*) genes are related and the associated enzymes are known to operate in either direction—reducing or oxidizing different S compounds. We found that *rdsrA* genes and *soxB* genes were expressed in the same samples, suggestive of active S cycling in the ETNP OMZ. These data provide potential thresholds for S cycling in OMZs that closely mimic recent predictions, and indicate that S cycling may be broadly relevant in OMZs.

## Introduction

Large areas of the ocean are characterized by low oxygen concentrations. These naturally occurring oxygen minimum zones (OMZs) are regions of pelagic ocean where dissolved oxygen (DO) concentrations are depleted below 20 μmol kg^-1^ due to microbial respiration (Helly and Levin, 2004; Paulmier and Ruiz-Pino, 2009; Gilly et al., 2013). OMZs are often associated with coastal and equatorial upwelling regions and the attendant high levels of microbial respiration driven by elevated primary production rates. OMZs are dominated by microbial communities, as much of the macrofauna of the pelagic ocean is unable to survive extended hypoxia (Diaz and Rosenberg, 2008; Gilly et al., 2013). At the same time, the total volume of OMZ waters is growing, their upper boundaries are vertically shoaling, and the degree of anoxia is intensifying within the cores of OMZs (Stramma et al., 2008; Gilly et al., 2013). These changes are collectively referred to as ‘ocean deoxygenation,’ and are driven by rising ocean temperatures that reduce oxygen solubility and increase water column stratification—both of which deplete DO concentrations at depth (Keeling et al., 2010).

The oxygen gradients found in OMZs create a chemically complex habitat that can sustain many types of microbial metabolism (Wright et al., 2012). While OMZs are well-known sites of nitrogen (N) cycling (Lam and Kuypers, 2011), recent work indicates that ‘cryptic’ sulfur (S) cycling occurs within the water column of the Eastern Tropical South Pacific Ocean (ETSP; Canfield et al., 2010). S metabolism is profoundly ancient, and genes related to S oxidation and reduction are dispersed throughout the Bacterial and Archaeal domains (Ghosh and Dam, 2009). S-cycling microorganisms, in turn, are distributed throughout a wide range of habitats—from marine sediments and eutrophic ponds, to animal guts and volcanic hot springs—where S exists in a range of chemical forms and oxidation states (Muyzer and Stams, 2008). However, S cycling was previously thought to occur in a relatively limited capacity in the ocean, specifically within anoxic basins such as the Black Sea, and ocean sediments (Canfield, 1989; Jørgensen et al., 1991). Active S cycling in the water column of open ocean OMZs has several implications for ocean biology and biogeochemistry, including the fact that hydrogen sulfide is toxic to large organisms (Lavik et al., 2009). The availability of different forms of S alters competitive and cooperative interactions among microbial groups and processes, and has an important bearing on organic matter remineralization and N cycling in OMZs (Johnston et al., 2014).

At the same time, several characteristic and abundant bacterial groups have been identified in OMZs and implicated in S cycling. This includes the γ-*proteobacteria* SUP05 and ARCTIC96BD-19 clades (Walsh et al., 2009), the δ-*proteobacteria* SAR324 (Swan et al., 2011; Sheik et al., 2014), and the Marine Group A (SAR406 clade; Wright et al., 2014). These groups typically comprise large percentages of OMZ microbial communities, although their specific niches are still ill-defined. Based on genomes and metagenomes, *SUP05*, *ARCTIC96BD-19*, and *SAR324* are now believed to have chemolithoautotrophic metabolisms that utilize sulfide and thiosulfate, as well as oxidized N, as sources of energy (Walsh et al., 2009; Canfield et al., 2010; Swan et al., 2011; Sheik et al., 2014). The oxidation of S using N (nitrate or nitrite) is known as ‘chemoautotrophic denitrification’ and is distinct from conventional, heterotrophic denitrification (oxidation of organic carbon using nitrate or nitrite), potentially with higher energy yields (Lam and Kuypers, 2011). This process plays a dual biogeochemical role by removing toxic sulfide (Lavik et al., 2009) while concurrently converting N to gaseous forms that may be ‘lost’ from the system. Marine Group A bacteria have also been implicated in S cycling, potentially reducing S in the water column (Wright et al., 2014). However, these broader groups are each made up many operational taxonomic units (OTUs) that are heterogeneously distributed within OMZs (Wright et al., 2012); whether these represent different ecotypes with distinct biogeochemical roles and ecological niches is also unknown.

We used quantitative PCR (qPCR) of 16S rRNA and functional genes to determine the distribution, relative abundance, and potential activity of these S-utilizing bacteria across the oxycline of the eastern tropical North Pacific OMZ. These samples were collected before cryptic S cycling was identified in the ETSP; however, archived DNA and cDNA offer the possibility of examining S-cycling processes based on gene expression patterns. In the ETSP, S was reduced and oxidized so rapidly that it was not easily detectable based on chemical measurements, yet transcriptomics provided a way to track S cycling (Canfield et al., 2010). We apply this approach along oxygen gradients present in the Eastern Tropical North Pacific (ETNP)—the ocean’s largest OMZ by volume (Paulmier and Ruiz-Pino, 2009)—to determine (1) whether S cycling may occur, and (2) how it is distributed along environmental gradients.

## Materials and Methods

### Sample Collection and DNA and RNA Extraction

**FIGURE 1:**
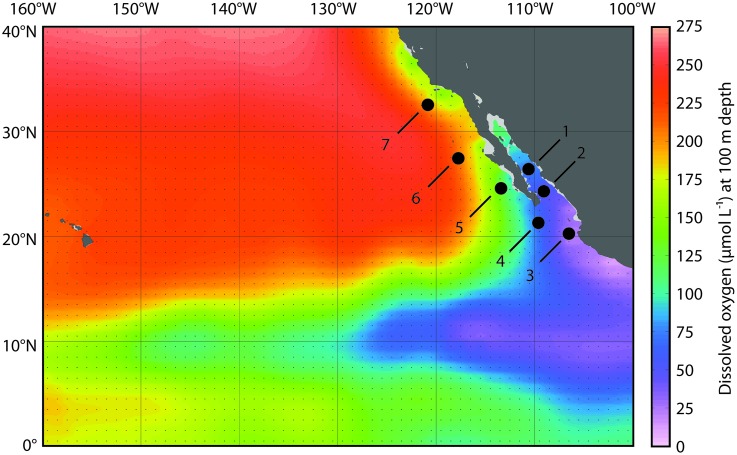
**Map of sampling stations in the eastern tropical North Pacific Ocean (ETNP)**. Station locations are plotted on dissolved oxygen (DO) concentrations (μmol L^-1^) at 100 m depth from the World Ocean Atlas (plotted in Ocean Data View).

Samples were collected aboard the *R/V New Horizon* in the Gulf of California (GOC) and ETNP in July–August 2008 (**Figure 1**). DO concentrations were measured using a Seabird oxygen sensor on the CTD and were corrected based on Winkler titrations (*r*^2^ = 0.997, *n* = 187 for the cruise). DNA/RNA sample collection depths were selected to capture the transition from hypoxic to suboxic conditions in the OMZ across 4–6 depths spaced every 10–20 m. Additional details of sampling are provided in Beman et al. (2012, 2013), Beman and Carolan (2013).

At each depth, two sets of 2 L samples were filtered through separate 25 mm diameter 0.2 μm Suppor filters (Pall Corporation, Port Washington, NY, USA) using a peristaltic pump. Filters were flash frozen in liquid nitrogen and stored at -80∘C until DNA and RNA extraction, with one set of filters dedicated to each. Details of DNA extraction were reported in Beman et al. (2012, 2013). Details of RNA extraction are reported in Beman and Carolan (2013). In brief, RNA was extracted using the Qiagen RNeasy kit following Church et al. (2005), and treated with DNase to remove carry-over DNA; cDNA was generated from extracted RNA using the Invitrogen SuperScript III Reverse Transcriptase kit (Life Technologies Corporation, Carlsbad, CA, USA) following the manufacturer’s instructions; remaining RNA was removed through incubation with RNase H (Invitrogen).

### Quantitative PCR and PCR

Quantitative PCR assays were performed on a Stragene MX3005P (Agilent Technologies, LA Jolla, CA, USA) using the following reaction chemistry: 12.5 μL SYBR Premix F (Epicentre Biotechnologies, Madison, WI, USA), 2 μL MgCl_2_, 1 μL of each primer, 0.25 μL AmpliTaq polymerase (Applied Biosystems, Life Technologies Corporation, Carlsbad, CA, USA), 40 ng μL^-1^ BSA, and 1 ng DNA in a final volume of 25 μL. Details of assays used are provided in **Table 1**. All assays were taken directly from the literature, and used standards that were synthesized using GeneArt Gene Synthesis (Life Technologies Corporation, Carlsbad, CA, USA). This included 16S genes from *Chromatiales* and SUP05 bacteria, as well as (reverse) dissimilatory sulfite reductase (*dsrA* and *rdsrA*) genes, and the thiosulfate-oxidizing enzyme system (*soxB*) gene. qPCR efficiencies and *R*^2^ for standard curves are also provided in **Table 1**. The efficiency of the *Chromatiales* assay is on the high end of acceptability for qPCR and likely stems from the use of a universal primer in this assay. For some assays, we tested only for presence/absence or expression/lack of expression within samples, and did not quantify gene abundances (see below). No efficiencies or *r*^2^ values are reported for these assays, as we used positive controls but did not generate a standard curve.

**Table 1:** **Details of quantitative PCR (qPCR) assays, including the primers used, the reference for the qPCR protocol, and the efficiency and *r*^2^ of standard curves**.

Gene	Forward primer (5′→ 3′)	Reverse primer (5′→ 3′)	qPCR protocol reference	Efficiency	*r*^2^
*Chromatiales* 16S rRNA	CHR986f AGCCCTTGACATCCTCGGAA	1392R ACGGGCGGTGTGTAC	Coolen and Overmann (1998)	125%	0.997
SUP05 16S rRNA	ba519F CAGCMGCCGCGGTAANWC	SUP051048R CCATCTCTGGAAAGTTCCGTCT	Zaikova et al. (2010)	99.2%	0.947
*dsrA*	dsr1F+ ACSCACTGGAAGCACGGCGG	dsrR GTGGMRCCGTGCAKRTTGG	Leloup et al. (2007)	95.2%	0.968
*rdsrA*	rdsr393f ACAGGCGGCATTACGTACC	rdsr810r AGTACGCTTTCCACCCATG	Lavik et al. (2009)		
*soxB*	soxB432F GAYGGNGGNGAYACNTGG	soxB1446 CATGTCNCCNCCRTGYTG	Swan et al. (2011)		

## Results and Discussion

### Oxygen Gradients

**FIGURE 2:**
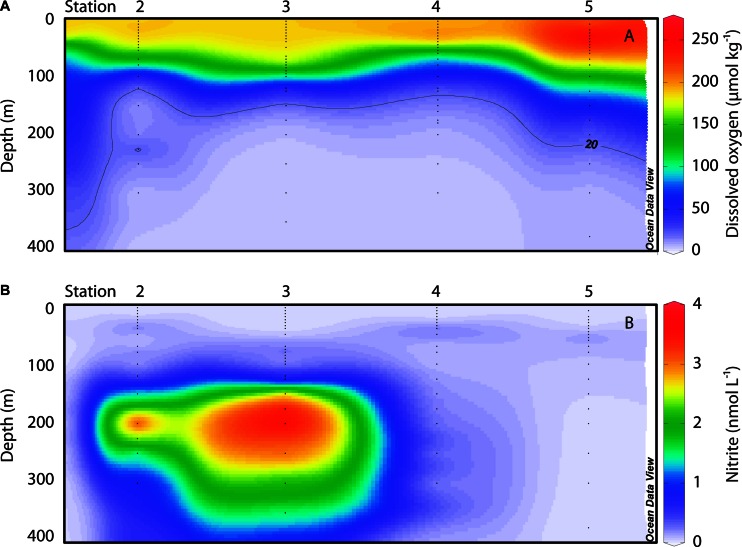
**Oceanographic sections of **(A)** DO (μmol kg^-1^) and **(B)** nitrite (nmol L^-1^)**. The location of individual sampling stations is indicated along the horizontal axis; vertical axes display depth down to 400 m; color scales show concentrations. Small black dots denote the depths of bottle samples collected for nitrite measurements, and discrete oxygen data for the same depths were extracted from the Winkler-corrected CTD data. Data were gridded in Ocean Data View.

Dissolved oxygen concentrations decline with depth in the ocean, and in OMZ regions they drop below 20 μmol kg^-1^. In the ETNP, these low DO values typically span several 100 m of the water column; they extend laterally from the coast of Mexico and Central America across the Pacific Ocean, north along the coast of Baja California, and also extend into the GOC (the body of water defined by the Baja California peninsula and mainland Mexico). We sampled a series of seven stations in the ETNP and GOC (**Figure 1**) that extend across this DO gradient: station 1 was located in the Carmen Basin of the GOC, station 2 was located near the mouth of the GOC, and station 3 was located in the core of the ETNP OMZ—where DO concentrations declined rapidly with depth and dropped to 20 μmol kg^-1^ by 111 m (**Figure 2**). Four additional stations extended north from the tip of Baja (station 4) to several 100 km off the coast of San Diego, California (station 7). We previously sequenced 16S rRNA genes using 454 pyrosequencing to characterize microbial communities in the upper water column and OMZ (Beman and Carolan, 2013); here we focus on the four stations (number 2–5) that have a well-developed OMZ.

The comparison among these stations provides systematic gradients in DO and nitrite (NO_2_^-^) concentrations with depth and between stations. DO reaches lower absolute values and the OMZ generally thickens, from north to south. OMZ waters (DO <20 μmol kg^-1^) were located at 91 m depth at station 2 (with an intrusion of water with slightly higher DO concentrations at 220–255 m), 111 m at station 3, 121 m at station 4, and 178 m at station 5. However, a recent review by Ulloa et al. (2012) distinguishes between typical OMZs and what they refer to as ‘anoxic marine zones’ (AMZs), where DO concentrations drop below 50 nM. They note these anoxic waters are also characterized by the accumulation of NO_2_^-^ in secondary nitrite maxima. In the ETNP, prominent secondary NO_2_^-^ maxima were observed at stations 2 and 3 in OMZ waters (**Figure 2**). NO_2_^-^ concentrations at these depths were an order of magnitude greater than in the primary nitrite maximum (PNM) at the base of the euphotic zone, reaching up to 4.18 μmol L^-1^ in the OMZ at station 2 and 4.24 μmol L^-1^ in the OMZ at station 3. This feature extended from ∼125–500 m at stations 2 and 3. In contrast, station 4 had low DO at depth, but NO_2_^-^ concentrations did not rise above 114 nM; at station 5, OMZ waters were found starting at 178 m, and the NO_2_^-^ concentration did not rise above 30 nM. Low DO waters define OMZs and allow for anaerobic N cycling to proceed; while it was previously thought that sulfate would only be exploited when oxidized N species were sufficiently depleted, recent evidence from the Eastern Tropical South Pacific OMZ indicates that a cryptic S cycle may occur in AMZs (Canfield et al., 2010; Ulloa et al., 2012). Our biogeochemical data indicate that stations 4 and 5 represent OMZs, whereas stations 2 and 3 represent AMZs.

### Abundance and Activity of Sulfur Cycling Bacterial Groups

Along these gradients we quantified specific groups and genes and also examined their activity: amplification of particular genes in DNA indicates the presence of a particular group or gene, whereas amplification in cDNA indicates that a particular group is actively growing or a particular gene is actively expressed. Our previous sequencing data indicated relatively high abundances of *Chromatiales* (Beman and Carolan, 2013), or purple-sulfur bacteria, a group capable of either chemo- or photo-trophic growth under anoxia (Imhoff, 2005). In anoxic aquatic ecosystems, *Chromatiales* are typically found in a narrow band where sufficient amounts of both sulfide and light are found; sulfide and light almost invariably display opposing gradients with depth, but can together sustain anoxygenic photosynthesis (Imhoff, 2001; Overmann, 2001).

**FIGURE 3:**
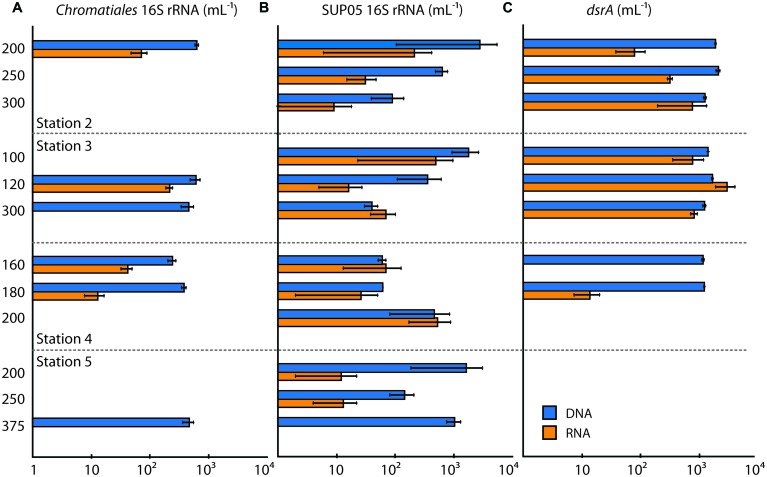
**Quantitative PCR (qPCR) data for **(A)***Chromatiales* 16S rRNA, **(B)** SUP05 16S rRNA, and **(C)***dsrA* genes and transcripts (expressed per mL)**. Station and depth of sample collected are shown, and gene or transcript abundances are displayed along the horizontal axes (log scale). Abundances in DNA are shown in blue, and abundances in RNA (i.e., cDNA) are shown in orange. Error bars denote SD of the triplicate measurement.

We quantified *Chromatiales* 16S rRNA to examine the distribution of these organisms with depth and from station to station. *Chromatiales* were readily detected in DNA and were present in the ETNP at all four stations, but did not appear to be highly active based on 16S rRNA genes in the cDNA fraction. *Chromatiales* 16S rRNA genes were detected at one depth at stations 2 and 5, and two depths at stations 3 and 4, and were most abundant at stations 2 and 3 (**Figure 3**). In contrast, 16S rRNA was not detected in most cDNA samples, and typically showed 1–2 orders of magnitude lower values than 16S rRNA genes in DNA. The ratio of 16S rRNA in cDNA versus DNA ranged from 0 to 0.36. *Chromatiales* 16S rRNA was most abundant at 120 m at station 3, but light levels at this depth—and in all other samples with *Chromatiales* 16S rRNA expression—were extremely low, and *Chromatiales* are most likely not photosynthetically active at these depths.

We also quantified *SUP05 Gammaproteobacteria*, which are abundant in hypoxic and anoxic waters worldwide (Walsh et al., 2009), and have been implicated in the ‘detoxification’ of sulfide plumes in OMZs/AMZs (Lavik et al., 2009; Schunck et al., 2013). In these cases, SUP05 and other S-oxidizing bacteria may ‘bloom’ at levels of up to 10^5^ cells ml^-1^. SUP05 were detected at all stations in the ETNP but were present in low abundance (<3000 16 rRNA genes ml^-1^; **Figure 3**). SUP05 were least abundant at station 4, and were present at higher levels at station 5—however, SUP05 were much less active at Station 5 than their numbers would suggest, and 16S rRNA was barely detectable in cDNA. At station 4, 16S rRNA profiles closely tracked 16S rRNA genes, showing comparable numbers and an increase with depth. In contrast, SUP05 were most abundant at stations 2 and 3—where there is a well-developed OMZ and AMZ—but were comparatively less active—with levels of 16S rRNA in cDNA that were 1–2 orders of magnitude lower than 16S rRNA genes in DNA. A key exception to this pattern was 300 m depth at station 3, where 16S rRNA in cDNA exceeded DNA values. This sample is characterized by low DO and high NO_2_^-^, and the relatively high levels of SUP05 activity are consistent with a role in chemoautotrophic denitrification—i.e., the oxidization of reduced S compounds using nitrate/NO_2_^-^ (Walsh et al., 2009).

**FIGURE 4:**
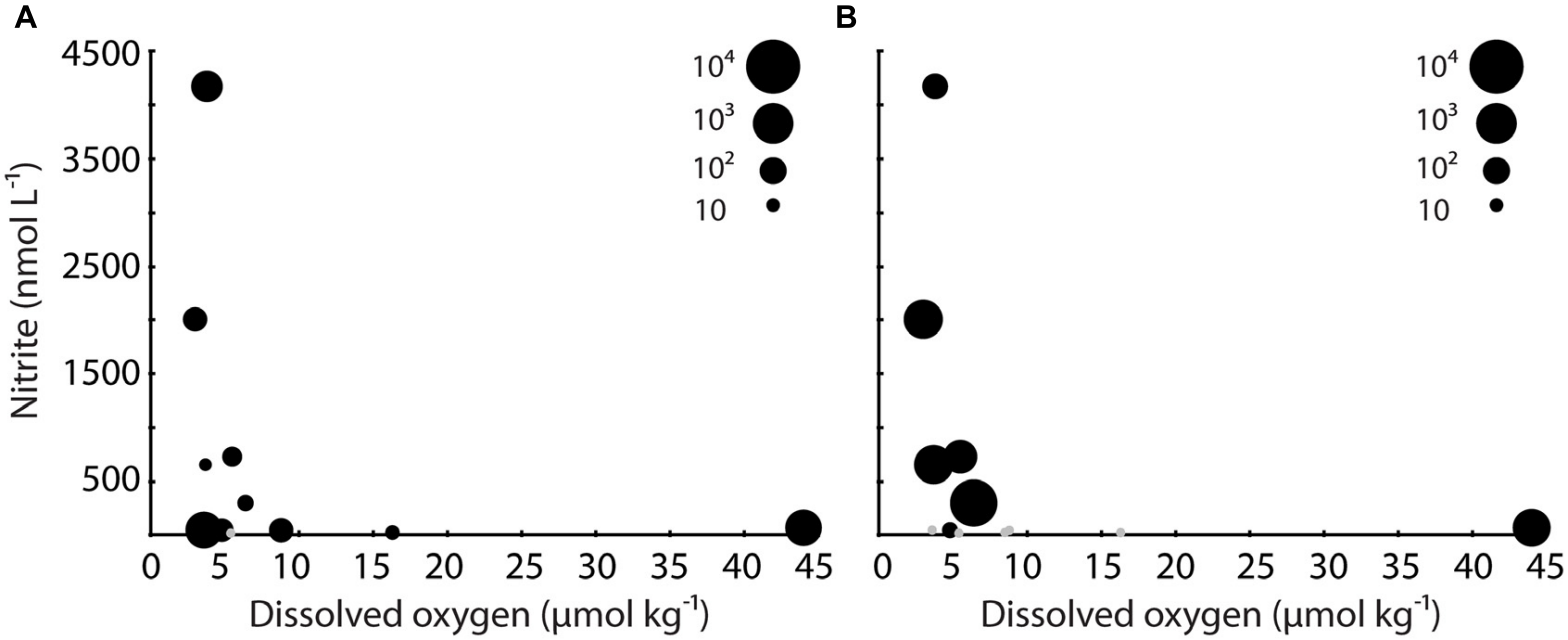
**Bubble plot of qPCR data for **(A)** SUP05 16S rRNA and **(B)***dsrA* plotted versus DO and nitrite**. The size of the bubbles is proportional to level of expression. Gray datapoints indicate that no expression was detected.

Despite these relatively high levels of 16S rRNA, the general pattern at stations 2 and 3 was declining SUP05 abundance and activity with increasing depth and NO_2_^-^ concentrations, and decreasing DO concentrations. This is consistent with surveys of the Black Sea and Baltic Sea, where SUP05-related organisms occur at highest abundance at the interface of oxic and anoxic waters (Glaubitz et al., 2013). There was a surprising amount of variation in DO and NO_2_^-^ concentrations where SUP05 were most abundant and active (at 200 m at station 2, 100 m at station 3, and 200 m at station 4; **Figure 4**). Stations 2 and 4 had low DO (<4 μmol kg^-1^) at 200 m, whereas DO concentrations where higher at 100 m at station 3; NO_2_^-^ concentrations were elevated at 200 m at station 4 (>4000 nM), and were lower at 200 m at station 4 and 100 m at station 3. This is consistent with the idea that SUP05 have considerable metabolic diversity and may tolerate a range of geochemical conditions (Walsh et al., 2009; Swan et al., 2011; Murillo et al., 2014). In the ETSP, for example, SUP05 are active throughout much of the water column, and their metagenomes show evidence for S and N metabolism, as well as mixotrophy (Murillo et al., 2014).

### Expression of Functional Genes involved in Sulfur and Nitrogen Cycling

To determine if a cryptic S cycle was possible in the ETNP OMZ, we quantified the DNA and cDNA gene copies for the *dsrA* gene, a hallmark of sulfate-reducing bacteria. The primers we used a specifically designed to target sulfate-reducing bacteria (Kondo et al., 2004), however, the *dsrA* gene is closely related to the *rdsrA* gene found within *Chlorobi*, *Alpha*-, *Beta*-, and *Gammaproteobacteria* (Loy et al., 2009). *dsrA* and *rdsrA* are known to act on sulfite, sulfate, and elemental S, allowing for diverse S metabolism (Ghosh and Dam, 2009), and may function in either the reductive or oxidative direction. *dsrA* expression is therefore indicative of S cycling, but in-depth omics and geochemical analyses are required to determine which S-cycling pathways are active at any given time.

Given the need for anaerobic conditions and the idea that NO_2_^-^ is indicative of true anoxia (Ulloa et al., 2012), we expected that *dsrA* expression would be greatest in OMZ waters with high NO_2_^-^ concentrations at stations 2 and 3. *dsrA* genes and transcripts were in fact most common and abundant at stations 2 and 3, supporting this hypothesis (**Figure 3**). *dsrA* genes were detected at 160 and 180 m at station 4 and were not detected at station 5. However, *dsrA* was expressed only at very low levels at 180 m at station 4, suggesting little to no activity outside of ‘AMZ’ conditions in the ETNP. These findings are similar to those of Stewart et al. (2012) in the ETSP, who found that *rdsrA*/*dsrA* genes were expressed throughout the OMZ, with the greatest proportional representation in the core of the OMZ. However, they identified ca. 90% of these transcripts as oxidative, *rdsrA* transcripts rather than reductive *dsrA* transcripts.

At station 2 in the ETNP, we found similar levels of *dsrA* genes throughout the water column (in DNA), but observed a pronounced increase in *dsrA* transcripts with increasing depth. This trend was not observed at station 3: instead, highest levels of expression occurred at 120 m depth, and *dsrA* was also expressed at 100 m, where DO was 44 μmol kg^-1^. There are several possible explanations for this, the first being that this is a spurious datapoint, as the remaining data all support the idea that *dsrA* is only expressed under low DO and elevated NO_2_^-^ (**Figure 3**). However, there are several alternative explanations. First, DO declined rapidly with depth at station 3, dropping from 190 μmol kg^-1^ at 85 m depth to<20 μmol kg^-1^ at 111 m. Along this sharp oxygen gradient, microorganisms may experience a range of DO concentrations through time as supply and demand vary, or as they are transported through the water column. Second, sulfate reduction could occur on particles within anoxic microsites. Earlier research suggested that this might be a common phenomenon (Shanks and Reeder, 1993). It is also possible that this particular sample—or some proportion of the expressed *dsrA* genes—reflects S oxidation, rather than reduction.

To gain greater insight into S oxidation, we used qPCR to detect *rdsrA* using primers that target *Alpha*- and *Gammaproteobacterial* S-oxidizers, such as *SUP05* and related lineages (Lavik et al., 2009). In our hands, we were not able to generate high quality PCR product (based on dissociation curves) and we report these data only in terms of presence/absence—i.e., was the gene detected and expressed, or not. *rdsrA* genes and transcripts were detected in all OMZ and AMZ samples collected in this study (**Table 2**). These findings are consistent with the presence and activity of SUP05 in these samples, as well as the large numbers of ARCTIC96BD-19 and SAR324 sequences recovered in our earlier work (Beman and Carolan, 2013). We also screened samples for the *soxB*, a gene that encodes for part of a thiosulfate oxidizing enzyme complex and is found in many bacterial lineages (Meyer et al., 2007; Ghosh and Dam, 2009). In contrast to *rdsrA*, *soxB* was only expressed at 200, 250, and 300 m depth at station 2 and 120 and 300 m at 3 (**Table 2**). These samples are all characterized by DO concentrations of 6.5 μmol kg^-1^ or lower and NO_2_^-^ concentrations of 299 nM or greater, suggesting that *soxB* expression is confined to AMZ waters in the ETNP. Our previous analysis of nitrate reductase genes (*narG* and *napA*) also showed that *narG, napA*, or both, were expressed where DO was low and NO_2_^-^ was elevated (presumably due to nitrate reduction to NO_2_^-^; Beman et al., 2013). S oxidation and conventional denitrification could occur independently of one another, but coincident expression of S oxidation and N reduction genes suggests that chemoautotrophic denitrification may occur in the ETNP.

**Table 2:** **Sample information and expression of functional genes involved in sulfur oxidation**.

Station	Depth (m)	DO (μmol kg^-1^)	NO_2_^-^ (nmol L^-1^)	*rdsrA* expression	*soxB* expression
2	200	3.8	4175	+	+
2	250	5.5	730	+	+
2	300	3.7	656	+	+
3	100	44	69	+	–
3	120	6.4	299	+	+
3	300	3.0	2008	+	+
4	160	8.8	45	+	–
4	180	4.8	46	+	–
4	200	3.6	47	+	–
5	200	16.3	24	+	–
5	250	8.5	25	+	–
5	375	5.4	19	+	–

*Plus sign indicates expression was detected*.

Assembled together, these data demonstrate that S cycling is active throughout the ETNP OMZ stations sampled in this study. The activity of SUP05 bacteria is consistent with anaerobic S oxidation in the OMZ, and expression of *dsrA*, *rdsrA*, and *soxB* indicates that several S-cycling pathways and processes may be active in the ETNP. Without definitive geochemical data, we cannot establish whether sulfate reduction and a cryptic S cycle are active in the ETNP, but this is one of at least three interpretations of our transcriptomic data. Recent analyses indicate that the clear separation between reductive *dsrA* gene sequences and oxidative *rdsrA* sequences allows for robust detection of these individual genes (Müller et al., 2014), and the *dsrA* primers that we used specifically target the reductive *dsrA* gene present in sulfate reducing bacteria (Kondo et al., 2004; Leloup et al., 2007; Müller et al., 2014). Alternatively, *dsrA* expression—along with *rdsrA,* and *soxB*—may capture S oxidation only. Given the expression patterns with depth and as a function of DO and nitrite, this is likely anaerobic S oxidation using oxidized N. This idea is supported by expression of nitrate reductase genes at the same depths (Beman et al., 2013). Chemoautotrophic denitrification has implications for N loss processes in the ETNP—which is still understudied compared with the ETSP and Arabian Sea, and is particularly relevant given the volume of the ETNP relative to the other OMZs. Finally, a third interpretation of our data is that they capture additional S-cycling processes beyond oxidation and reduction. For example, Schunck et al. (2013) raise the possibility of S disproportionation in the ETSP based on the joint expression of *dsrA*, *soxB*, and other S-cycling genes. Based on the SAR324 metagenome, Sheik et al. (2014) discuss lithoheterotrophy as a potentially important form of metabolism, and the SUP05 metagenome recovered from the ETSP also supports this idea (Murillo et al., 2014). These findings add layers of complexity to OMZ S cycling, such that significant geochemical and ‘omics research will be necessary to unravel the multiple forms of S metabolism and their connections to C and N cycling. Our data represent the first piece of evidence for S metabolism in the ETNP OMZ, and suggest that S cycling is prevalent in the ocean’s OMZs. Determining the significance of S cycling in terms of rates, lateral extent, and depth distribution in the ETNP will be key for understanding the biogeochemical and ecological implications of water column S cycling in the ETNP, and globally.

## Author Contributions

JMB designed the study; JMS and JMB collected the samples; MTC performed the laboratory work; MTC and JMS contributed new reagents and analytical tools; MTC and JMB analyzed the data; MTC wrote the paper; JMS and JMB edited the paper.

## Conflict of Interest Statement

The author declares that the research was conducted in the absence of any commercial or financial relationships that could be construed as a potential conflict of interest.

## References

[B1] Beman J. M. Carolan M. T. (2013). Deoxygenation alters bacterial diversity and community composition in the ocean’s largest oxygen minimum zone. *Nat. Commun.* 4 2705 10.1038/ncomms3705 24162368

[B2] Beman J. M. Popp B. N. Alford S. E. (2012). Quanitifcaiton of ammonia oxidation rates and ammnia oxidizing archaea and bacteria at high resolution in the Gulf of California and the eastern tropical North Pacific. *Limnol. Oceanogr.* 57 711–726. 10.4319/lo.2012.57.3.0711

[B3] Beman J. M. Shi J. L. Popp B. N. (2013). Nitrite oxidation in the upper water column and oxygen minimum zone of the eastern tropical North Pacific Ocean. *ISME J.* 7 2192–2205. 10.1038/ismej.2013.96 23804152 PMC3806268

[B4] Canfield D. E. (1989). Sulfate reduction and oxic respiration in marine sediments: implications for organic carbon preservation in euxinic environments. *Deep Sea Res. A* 38 121–138. 10.1016/0198-0149(89)90022-8 11542177

[B5] Canfield D. E. Stewart F. J. Thamdrup B. Brabandere L. D. Dalsgaard T. Delong E. F. (2010). A cryptic sulfur cycle in the oxygen minimum zone waters off the chilean coast. *Science* 330 1375–1378. 10.1126/science.1196889 21071631

[B6] Church M. J. Short C. M. Jenkins B. D. Karl D. M. Zehr J. P. (2005). Temporal patterns of nitrogenase gene (nifH) expression in the oligotrophic North Pacific Ocean. *Appl. Environ. Microbiol.* 71 5362–5370. 10.1128/AEM.71.9.5362-5370.2005 16151126 PMC1214674

[B7] Coolen M. J. Overmann J. (1998). Analysis of subfossil molecular remains of purple sulfur bacteria in a lake sediment. *Appl. Environ. Microbiol.* 64 4513–4521. 9797316 10.1128/aem.64.11.4513-4521.1998 PMC106678

[B8] Diaz R. J. Rosenberg R. (2008). Spreading dead zones and consequences for marine ecosystems. *Science* 321 926–929. 10.1126/science.1156401 18703733

[B9] Ghosh W. Dam B. (2009). Biochemistry and molecular biology of lithotrophic sulfur Oxidation by taxonomically and ecologically diverse bacteria and archaea. *FEMS Microbiol. Rev.* 33 999–1043. 10.1111/j.1574-6976.2009.00187.x 19645821

[B10] Gilly W. F. Beman J. M. Litvin S. Y. Robinson B. M. (2013). Oceanographic and biological effects of shoaling of the oxygen minimum zone. *Ann. Rev. Mar. Sci.* 5 393–420. 10.1146/annurev-marine-120710-100849 22809177

[B11] Glaubitz S. Kießlich K. Meeske C. Labrenz M. Jürgens K. (2013). SUP05 dominates the gammaproteobacterial sulfur oxidizer assemblages in pelagic redoxclines of the central Baltic and Black Seas. *Appl. Environ. Microbiol.* 79 2767–2776. 10.1128/AEM.03777-12 23417000 PMC3623164

[B12] Helly J. J. Levin L. A. (2004). Global distribution of naturally occurring marine hypoxia on continental margins. *Deep Sea Res. I* 51 1159–1168. 10.1016/j.dsr.2004.03.009

[B13] Imhoff J. F. (2001). True marine and halophilic anoxygenic phototrophic bacteria. *Arch. Microbiol.* 178 243–254. 10.1007/s002030100326 11685368

[B14] Imhoff J. F. (2005). “Chromatiales ord. nov,” in *Bergey’s Manual of Systematic Bacteriology,”* 2nd Edn, eds Brenner D. J. Krieg, N. R. Staley J. T., Garrity G. M.(New York, NY: Springer).

[B15] Johnston D. Gill B. Masterson A. Beirne E. Casciotti K. Knapp A. (2014). Placing an upper limit on cryptic marine sulphur cycling. *Nature* 513 530–533. 10.1038/nature13698 25209667

[B16] Jørgensen B. B. Fusing H. Wirsen C. O. Jannasen H. W. (1991). Sulfide oxidation in the anoxic Black Sea chemocline. *Deep Sea Res. A* 38 S1083–S1103. 10.1016/S0198-0149(10)80025-1

[B17] Keeling R. F. Kurtzinger A. Gruber N. (2010). Ocean deoxygenation in a warming world. *Ann. Rev. Mar. Sci.* 2 199–229. 10.1146/annurev.marine.010908.163855 21141663

[B18] Kondo R. Nedwell D. B. Purdy K. J. Silva S. Q. (2004). Detection and enumeration of sulphate-reducing bacteria in estuarine sediments by competitive PCR. *Geomicrobiol. J.* 21 145–157. 10.1080/01490450490275307

[B19] Lam P. Kuypers M. M. M. (2011). Microbial nitrogen cycling processes in oxygen minimum zones. *Annu. Rev. Marine Sci.* 3 317–345. 10.1146/annurev-marine-120709-142814 21329208

[B20] Lavik G. T. Stuührmann V. Brüchert A. Van der Plas V. Mohrholz P. Lam M. (2009). Detoxification of sulphidic African shelf waters by blooming chemolithotrophs. *Nature* 457 581–584. 10.1038/nature07588 19078958

[B21] Leloup J. Loy A. Knab N. J. Borowski C. Wagner M. Jørgensen B. B. (2007). Diversity and abundance of sulfate-reducing microorganisms in the sulfate and methane zones of a marine sediment, Black Sea. *Environ. Microbiol.* 9 131–142. 10.1111/j.1462-2920.2006.01122.x 17227418

[B22] Loy A. Duller S. Baranyi C. Mushmann M. Ott J. Shanon J. (2009). Reverse dissimilatory sulfite reductase as a phylogenetic marker for a subgroup of sulfur oxidizing prokaryotes. *Environ. Microbiol.* 11 289–299. 10.1111/j.1462-2920.2008.01760.x 18826437 PMC2702494

[B23] Meyer B. Imhoff J. F. Kuever J. (2007). Molecular analysis of the distribution and phylogeny of the soxB gene among sulfur-oxidizing bacteria. *Environ. Microbiol.* 11 2957–2977. 10.1111/j.1462-2920.2007.01407.x 17991026

[B24] Müller A. L. Kjeldsen K. U. Rattei T. Pester M. Loy A. (2014). Phylogenetic and environmental diversity of DsrAB-type dissimilatory (bi) sulfite reductases. *ISME J.* 10.1038/ismej.2014.208 [Epub ahead of print]. PMC4351914 25343514

[B25] Murillo A. A. Ramírez-Flandes S. DeLong E. F. Ulloa O. (2014). Enhanced metabolic versatility of planktonic sulfur-oxidizing γ-proteobacteria in an oxygen-deficient coastal ecosystem. *Aquat. Microbiol.* 1 18 10.3389/fmars.2014.00018

[B26] Muyzer G. Stams A. J. M. (2008). The ecology and biotechnology of sulphate-reducing bacteria. *Nat. Rev. Microbiol.* 6 441–454. 10.1038/nrmicro1892 18461075

[B27] Overmann J. (2001). Diversity and ecology of phototrophic sulfur bacteria. *Microbiol. Today* 28 116–119.

[B28] Paulmier A. Ruiz-Pino D. (2009). Oxygen minimum zones in the modern ocean. *Prog. Oceanogr.* 80 113–128. 10.1016/j.pocean.2008.08.001

[B29] Schunck H. Lavik G. Desai D. K. Großkopf T. Kalvelage T. Löscher C. R. (2013). Giant hydrogen sulfide plume in the oxygen minimum zone off Peru supports chemolithoautotrophy. *PLoS ONE* 8:e68661 10.1371/journal.pone.0068661 PMC3749208 23990875

[B30] Shanks A. L. Reeder M. L. (1993). Reducing microzones and sulfide production in marine snow. *Mar. Ecol. Prog. Ser.* 96 43–47. 10.3354/meps096043

[B31] Sheik C. S. Jain S. Dick G. J. (2014). Metabolic flexibility of enigmatic SAR324 revealed through metagenomics and metatranscriptomics. *Environ. Microbiol.* 16 304–317. 10.1111/1462-2920.12165 23809230

[B32] Stewart F. J. Ulloa O. DeLong E. F. (2012). Microbial metatranscriptomics in a permanent marine oxygen minimum zone. *Environ. Microbiol.* 14 23–40. 10.1111/j.1462-2920.2010.02400.x 21210935

[B33] Stramma L. Johnson G. C. Sprintall J. Mohrholz V. (2008). Expanding oxygen minimum zones in the tropical oceans. *Science* 320 655–658. 10.1126/science.1153847 18451300

[B34] Swan B. K. Martinze-Garcia M. Preston C. M. Woyke A. T. Lamy D. Reinthaler T. (2011). Potential for chemolithoautotrophy among ubiquitous bacteria lineages in the dark ocean. *Science* 333 1296–1300. 10.1126/science.1203690 21885783

[B35] Ulloa O. Canfield D. E. DeLong E. F. Letelier R. M. Stewart F. J. (2012). Microbial oceanography of anoxic oxygen minimum zones. *Proc. Natl. Acad. Sci. U.S.A.* 109 15996–16003. 10.1073/pnas.1205009109 22967509 PMC3479542

[B36] Walsh D. A. Zaikova E. Howes C. G. Sung Y. C. Wright J. J. Tringe S. G. (2009). Metagenome of a versatile chemolithoautotroph from expanding oceanic dead zones. *Science* 326 578–582. 10.1126/science.1175309 19900896

[B37] Wright J. J. Knowar K. M. Hallam S. J. (2012). Microbial ecology of expanding oxygen minimum zones. *Nat. Rev. Microbiol.* 10 381–394. 10.1038/nrmicro2778 22580367

[B38] Wright J. J. Mewis K. Hanson N. W. Konwar K. M. Maas K. R. Hallam S. J. (2014). Genomic properties of Marine Group A bacteria indicate a role in the marine sulfur cycle. *ISME J.* 8 455–468. 10.1038/ismej.2013.152 24030600 PMC3906813

[B39] Zaikova E. Walsh D. A. Stilwell C. P. Mohn W. W. Tortell P. D. Hallam S. J. (2010). Microbial community dynamics in a seasonally anoxic fjord: Saanich Inlet, British Columbia. *Environ. Microbiol.* 12 172–191. 10.1111/j.1462-2920.2009.02058.x 19788414

